# Co-Induction of ULK-1 and AHSP mRNAs in Erythroid Precursor Cells Isolated From a Sirolimus-Treated β-Thalassemia Patient: A Case Report Study

**DOI:** 10.3389/bjbs.2025.14311

**Published:** 2025-06-27

**Authors:** Matteo Zurlo, Alessia Finotti, Maria Rita Gamberini, Roberto Gambari

**Affiliations:** ^1^ Department of Life Sciences and Biotechnology, University of Ferrara, Ferrara, Italy; ^2^ Center “Chiara Gemmo and Elio Zago” for the Research on Thalassemia, University of Ferrara, Ferrara, Italy

**Keywords:** β-thalassemia, sirolimus, autophagy, Ulk-1, AHSP

## Abstract

**Introduction:**

The β-thalassemias are inherited genetic disorders affecting the hematopoietic system and caused by mutations of the adult β-globin gene, leading to low or absent production of adult hemoglobin. In addition, an excess of free α-globin is associated with ineffective erythropoiesis. In fact, the free α-globin molecules are prone to precipitate, causing toxicity to the erythroid cells, and interference with red cell maturation. In order to counteract the detrimental effects of the excess of α-globin, two pathways might be activated in β-thalassemia erythroid cells, i.e. Unc-51-like kinase 1 (Ulk-1)-mediated induction of autophagy and increased expression of the α-hemoglobin stabilizing protein (AHSP).

**Case Presentation:**

The studied case was a male transfusion dependent TM (Thalassemia Major) patient, aged 43 years, with a β^0^39/β^+^IVSI-110 genotype (XmnI polymorphism: -/-), starting the first blood transfusion when he was 5 months old, and participating to the NCT03877809 (Sirthalaclin) clinical trial.

**Methods:**

Expression of AHSP and Ulk-genes in Erythroid precursor cells (ErPCs) was studied by Reverse transcription (RT)-qPCR and Western blotting ErPCs were isolated from the propositus after 90 and 180 days of treatment with sirolimus.

**Results and Discussion:**

This study demonstrates for the first time that increase in the production of γ-globin2 mRNA and HbF in ErPCs from a patient with β-thalassemia treated with sirolimus might be associated with co-induction of Ulk-1 and AHSP genes.

## Introduction

The β-thalassemias are inherited genetic disorders affecting the hematopoietic system and caused by more than 350 mutations of the adult β-globin gene [1, 2]. These genetic mutations cause low or absent production of adult hemoglobin (HbA) [1]. In addition, a clinical parameter affecting the pathophysiology of erythroid cells in β-thalassemia is the excess of free α-globin [3, 4], that is caused by the lack or absent production of the β-globin chains to bind with. This deeply affects the production of the HbA (α_2_β_2_) tetramer under normal physiological conditions [1]. This is clinically relevant, since the free α-globin molecules are prone to precipitate, causing toxicity to the erythroid cells, interference with red cell maturation [5, 6], and ineffective erythropoiesis, as recently reviewed [7]. Accordingly, several concurrent evidences show that decreased expression of α-globin genes is beneficial for β-thalassemia patients [8, 9]. For instance, using the CRISPR-Cas9 approach, several authors have confirmed that downregulation of α-globin is associated with a milder phenotype of β-thalassemia [10, 11]. This conclusion was also supported in transgenic mice mimicking β-thalassemia [12].

In this context, the activation of two pathways has been described in erythroid cells, able to reduce the excess of free α-globin and/or counteracting its cytotoxicity. The first pathway is the activation of Unc-51-like kinase 1 (Ulk-1) dependent autophagy, as proposed by Lechauve et al. (2019) [13] who found that loss of Ulk-1 gene in β-thalassemic mice reduces autophagic clearance of α-globin in red blood cell precursors and exacerbates the disease phenotype. Systemic treatment with the mTORC1 inhibitor rapamycin reduces α-globin precipitates and lessens the pathology symptoms in β-thalassemic mice via an Ulk1-dependent pathway [13]. Similarly, rapamycin reduces free α-globin accumulation in erythroblasts derived from CD34^+^ cells of β-thalassemic individuals [14, 15]. In agreement with these results, Zurlo et al. (2023) found that rapamycin induces autophagy and increased expression of Ulk-1 mRNA in a cohort of β-thalassemia patients treated with low dosages of sirolimus and participating to the Sirthalaclin NCT03877809 clinical trial [15]. This is important, considering that the autophagic process is able to reduce the excess of free α-globin by activation of a proteasome dependent detoxification process [13].

The second pathway is associated to the biological activity of the α-hemoglobin stabilizing protein (AHSP), a chaperone highly expressed in erythroid cells and involved in counteracting α-globin precipitation and related cytotoxicity [16–18]. In this respect, Zurlo et al. (2024) have published a study demonstrating high expression of AHSP gene in β-thalassemia [19]. Interestingly, AHSP mRNA production is increased in β-thalassemia patients treated with sirolimus and participating to the NCT 03877809 clinical trial (Sirthalaclin). Design and key results of this trial have been described elsewhere [20, 21].

No information is available about the possible co-activation of the Ulk1-and AHSP-dependent pathways in sirolimus-treated erythroid cells from β-thalassemia patients. We here present biochemical and molecular analyses of a β-thalassemia patient to determine whether expression of Ulk-1 and AHSP genes can be co-activated.

## Case Description

The studied case was a male transfusion dependent TM (Thalassemia Major) patient, aged 43 years, with a β^0^39/β^+^IVSI-110 genotype, starting the first blood transfusion when he was 5 months old, and participating to the NCT03877809 (Sirthalaclin) clinical trial. Clinical information regarding the patient is reported in Table 1; information on the treatment with sirolimus (1 mg/day) can be found in Zuccato et al. [21].

**TABLE 1:** Clinical parameters of the patient at the time of recruitment to the NCT03877809 trial.

Clinical parameters	Comments/ongoing therapies
Genotype	β-globin gene: β^0^39/β^+^IVSI-110 XmnI polymorphism: -/-
A. General parameters	
Regular transfusion therapy	From the age of 5 months (December 1979)
Chelation therapy	Desferioxamine sc (30 mg/kg 6/7) from the age of 18 months up to 25/12/2017; later, Deferasirox FC per os (13,42 mg/kg 7/7)
Iron overload	Mean annual serum ferritin levels ranged from 2164 ng/mL in 2011 to 1,221 ng/mL in 2019. Iron status evaluations were regularly performed (every 18 months) from 2010 to 2019 by MRI-T2*; iron accumulations were reported normal in the heart, normal/mild in the liver; at last examination (on 31/1/2019) cardiac T2* was 40 ms, liver iron concentration was 4,21 mg/g liver dry tissue
Splenomegaly	Mild enlargement (longitudinal diameter 15,5 cm)
B. Clinical complications	
Chronic Hepatitis C (genotype 2)	Long term responder to the anti-viral therapy with Peg-Interferon + Ribavirin performed in 2012
Bone diseases	Osteopenia of lumbar spine (2009) Platyspondily of thoracic spine (2015)
Vitamin D deficiency	Supplementation with Cholecalciferol, 1000 IU/day, starting from 2012
Ectopic extramedullary hematopoiesis	Three paravertebral masses were diagnosed in 2017; maximum diameter 2,2 cm, stable at follow-up in 2019

Information on biochemical and molecular parameters following sirolimus treatment is reported in Supplementary Table S1. Accordingly with previously reported results [21], increase of γ-globin mRNA (and HbF production) occurred in ErPC isolated from this patient after 90–180 days of treatment with sirolimus. This was associated with a decrease in free α-globin chains [21], and levels of bilirubin, soluble transferrin receptor and ferritin (Supplementary Table S1). Transfusion demand evaluated at the end of treatment (360 days) decreased by 12.48% (for information on the employed methodology see Zuccato et al., 2021) [21]. The range of sirolimus accumulated in blood was 1.5 (V6, 90 days of treatment) and 4.6 (V8, 180 days of treatment) pg/mL; no major side effects and no alteration of the immunophenotype were noted, according with elsewhere reported results [21].

**FIGURE 1:**
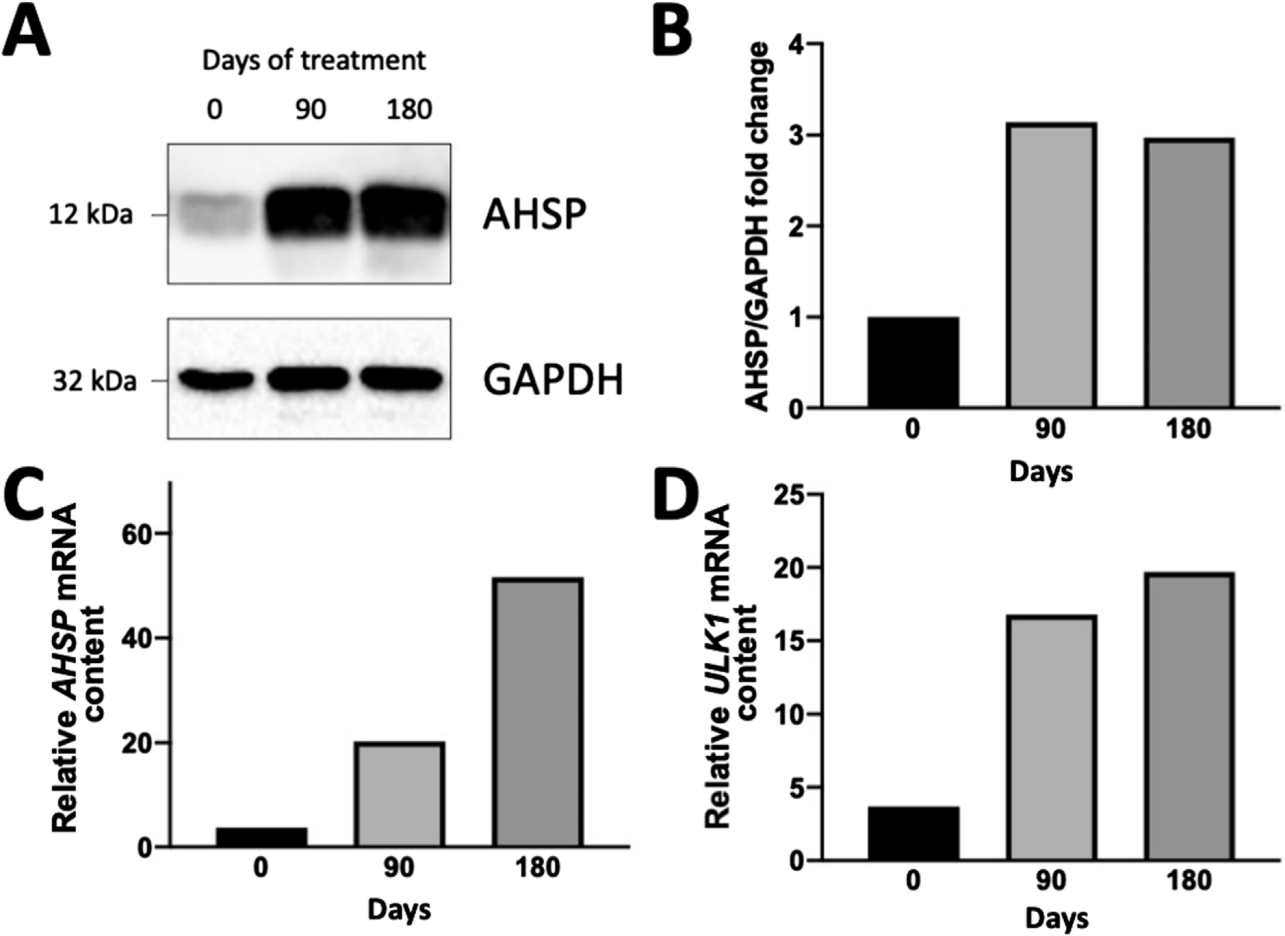
Expression of AHSP **(A–C)** and Ulk-1 **(D)** genes in ErPCs isolated from the propositus after 90 and 180 days of treatment with sirolimus as elsewhere reported [21]. **(A,B)** Western blotting analysis. Autoradiograms (the uncut version of the gels is shown in Supplementary Figure S1) are shown in panel **(A)**; the densitometric analysis is shown in panel **(B)**. **(C,D)** RT-qPCR analysis showing the relative content of AHSP **(C)** and Ulk-1 **(D)** mRNAs (internal control: GAPDH). The protocols and antibodies used for the Western blotting shown in **(A)** have been reported in Zurlo et al. (2024) [19]. The protocols and PCR primers for the RT-qPCR analyses shown in **(C,D)** have been reported in Zurlo et al. (2023) [15] (for Ulk-1 mRNA) and Zurlo et al. (2024) [19] (for AHSP mRNA).

In the Erythroid Precursor Cells (ErPcs) isolated from this patient, the expression of Ulk-1 was higher than 10 fold after 90 days treatment with 1 mg/day sirolimus, compared with Ulk-1 expression of ErPCs isolated from the same patient before the initial treatment with sirolimus and similar to ErPCs from other patients participating to the NCT03877809 clinical trial [15, 21]. In order to verify whether the AHSP gene was upregulated in these ErPCs (upregulating Ulk-1) [15], the expression of AHSP was evaluated by RT-qPCR and by Western blotting. The results are shown in Figure 1.

The results presented in Figure 1 demonstrate that ErPCs isolated at V6 (after 90 days of *in vivo* treatment with sirolimus) and V8 (after 180 days of *in vivo* treatment with sirolimus) accumulate a much larger amount of AHSP protein with respect to V2 ErPCs (Figures 1A,B). These data are completely in agreement with the RT-qPCR data shown in Figure 1C, that indicates that the AHSP RNA content at V6 and V8 is 20-fold and 48-fold higher compared to V2, respectively. The data concerning the Ulk-1 mRNA expression are shown in Figure 1D, that confirms that the Ulk-1 mRNA content at V6 and V8 is 16-fold and 18-fold higher with respect to V2, respectively. These data were reproducibly confirmed when different housekeeping sequences (β-actin, GAPDH, RPL13A) were employed as internal controls in the RT-qPCR analyses. This study demonstrates that increase in the production of γ-globin mRNA and HbF in erythroid precursor cells (ErPCs) from patients with β-thalassemia treated with sirolimus [21] might be associated with co-induction of Ulk-1 and AHSP genes.

**FIGURE 2:**
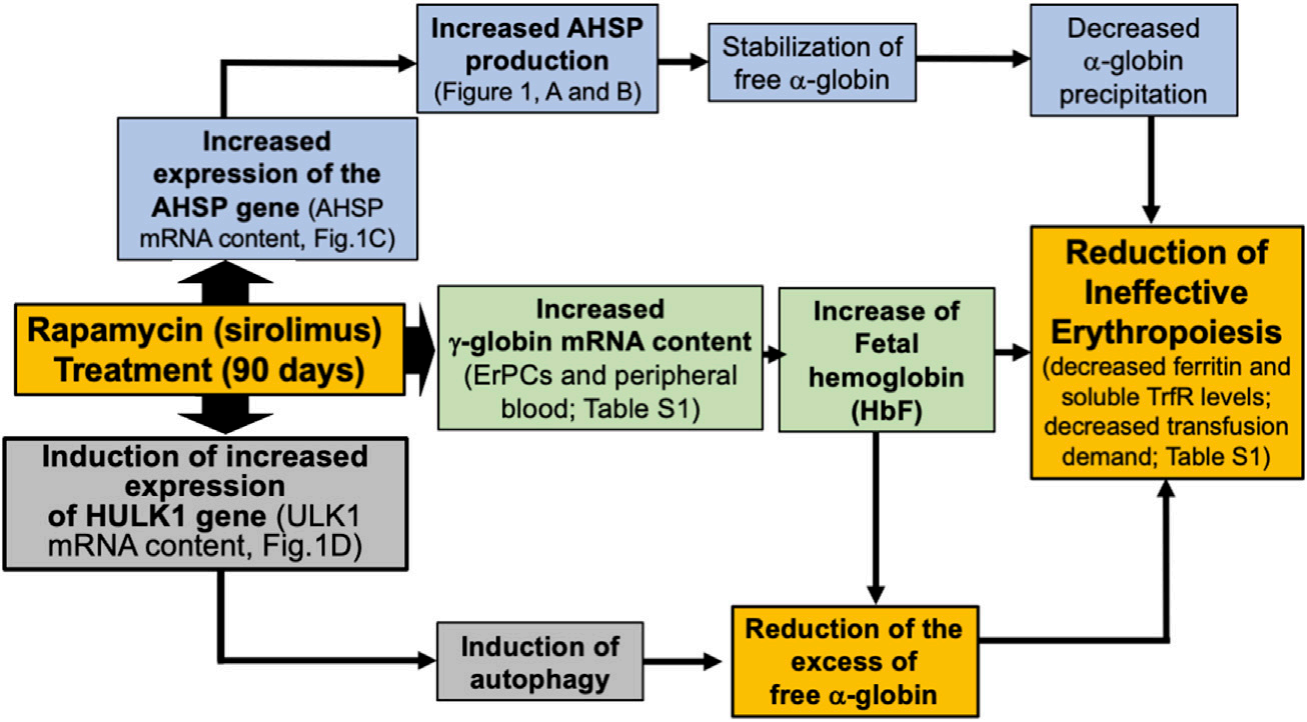
Pictorial representation of the proposed mechanism of action of sirolimus (rapamycin), based on the results of the present Case Report Study. Sirolimus induces an increase in the expression of γ-globin genes (Supplementary Table S1) and an increased HbF production [21]. Furthermore, sirolimus induced an increase in the expression of the AHSP gene (Figures 1A–C), possibly leading to stabilization of free α-globin and an increase in the expression of the Ulk-1 gene (Figure 1D), possibly leading to the induction of autophagy. Induction of fetal hemoglobin and autophagy co-operate in reducing excess free α-globin. AHSP-dependent stabilization of free α-globin and Ulk-1/autophagy-dependent reduction of excess free α-globin might contribute to the reduction of ineffective erythropoiesis.

Accordingly, sirolimus decreases the excess free α-globin [19, 21]. Therefore, the activity of sirolimus *in vivo* could occur through the induction of HbF and γ-globin genes, the activation of autophagy, associated with the upregulation of Ulk-1, the upregulation of AHSP, and the decrease in excess α-globin and inefficient erythropoiesis (as summarized in Figure 2).

## Discussion and Clinical Assessment

The relevance of the results of this Case Report Study is that the novel data presented support the conclusion that the expression of Ulk-1 and AHSP can be co-upregulated by sirolimus in ErPCs from β-thalassemia patients.

A limitation of the data presented in this Case Report, is that they originate from a single-patient study; therefore, our pilot study needs validation in larger cohorts of β-thalassemia patients. Accordingly, our results are expected to stimulate RT-qPCR analysis on ErPCs isolated from the other patients participating to the Sirthalaclin NCT03877809 and Thala-Rap NCT 04247750 clinical trials [15, 20, 21]. Our results support the concept that increased HbF and γ-globin mRNA content, expression of Ulk-1 and autophagy, increased AHSP gene expression should be considered as key end points for future clinical studies.

Finally, our study might stimulate research efforts focusing on the molecular basis of the co-expression of the Ulk-1 and AHSP genes. In this context, a very interesting possibility for autophagy activation in erythroid cells is based on the modulation of GATA-1, a master regulator of erythropoiesis, also regulating autophagy [22–24]. In this respect, Kang et al. (2012) were able to demonstrate, using Chromatin Immunoprecipitation (ChIP) assays, that GATA-1 directly interacts with regulatory sites of many autophagy genes [24]; these molecular approaches demonstrated that GATA-1 regulates autophagy [24]. Finally, GATA-1 regulates the expression of AHSP gene [25]. Further studies are required to verify this hypothesis in β-thalassemia erythroid cells.

### Genomic Report

Gene analysis demonstrated that this patient is compound heterozygous for β^0^39 and β^+^IVSI-110. In addition, sequencing data indicate that this patient was (−/−) for the XmnI polymorphism.

### Clinical Implications

Co-induction of Ulk-1 and AHSP genes in individuals with β-thalassemia might be associated with improved ineffective erythropoiesis, which is likely to result from a decrease of free α-globin. Expression of Ulk-1 and AHSP genes should be considered among the outcomes of treatment of β -thalassemia patients with HbF inducers, such as sirolimus. Regarding the effects of sirolimus on biochemical markers of ineffective erythropoiesis, Supplementary Table S1 shows a reduction in total bilirubin, soluble transferrin receptor, and ferritin levels after 90 and 180 days of sirolimus treatment. Further studies are required to determine whether these effects are dependent from the co-induction of Ulk-1 and AHSP.

### Conclusion

This study demonstrates for the first time that the elsewhere reported increase in the production of γ-globin mRNA and HbF in ErPCs from patients with β-thalassemia treated with 1 mg/day sirolimus (21) might be associated with co-induction of Ulk-1 and AHSP genes. Notably, sirolimus might decrease in this patient the excess of free α-globin and inefficient erythropoiesis through (a) the induction of HbF and γ-globin genes, (b) the activation of autophagy, associated with the upregulation of Ulk-1, and (c) the upregulation of AHSP. These effects are clinically relevant and suggest that further treatments with HbF inducers might be considered for this patient.

### Summary Sentence

The description of this case represents a significant advance in biomedical science by highlighting that sirolimus (rapamycin) treatment might be associated with co-induction of Ulk-1 and AHSP genes. This study expands our understanding of thalassemia syndromes and its treatment with the HbF inducer sirolimus.

## Data Availability

Data is provided within the article or Supplementary Material. Additional data, including additional clinical data and technical data on RT-qPCR, will be shared with other researchers upon reasonable request to the corresponding authors, maintaining the confidentiality of patient information.
