# Supplementary material for: Co-Induction of ULK-1 and AHSP mRNAs in Erythroid Precursor Cells Isolated From a Sirolimus-Treated β-Thalassemia Patient: A Case Report Study
Source: Br J Biomed Sci. 2025 Jun 27;82:14311. doi: 10.3389/bjbs.2025.14311 (PMC12245733; doi:10.3389/bjbs.2025.14311)
Supplement: Supplementary file 1 [file DataSheet1.docx]

**SUPPLEMENTARY MATERIALS**

**Supplementary Table S1**

**TABLE S1.** Variation of biochemical and molecular parameter in the patient after sirolimus treatment (*)

**Biochemical or molecular parameter V2 V6 V8**

ErPC γ-globin mRNA (content relative to V2) 1 7.10 6.50

Variation of bilirubin levels (mg/dL) 2.68 1.92 1.62

Variation of ferritin levels (mg/dL) 1221 1213 1191

Variation of soluble transferrin receptor levels (mg/L) 3.94 2.71 2.60

(*) Adapted from Zuccato et al. [2022]. Methods are described in Zuccato et al. [2022]. Sirolimus treatment was for 90 days (V6, visit 6) and 180 days (V8, visit 8).

**Supplementary Table S2.**

**TABLE S2.** List of primers and probes with related sequences used to perform RT-qPCR analyses.

**Primers and Probes Sequences**

primer forward ULK1 5′-CTACCTGGTTATGGAGTACTGC-3′

primer reverse ULK1 5′-GGAAGAGCCTGATGGTGTC-3′

probe ULK1 5′-FAM-CGACTACCT/ZEN/GCACGCCATGC-BFQ-3′

primer forward AHSP 5′-GAGACATATACAGCCTGTTAGACC-3′

primer reverse AHSP 5′-GAGGATCATTGAAGACCTGCT-3′

probe AHSP 5′-FAM -ATGAGATCCTTATTGGCCTTAAGAAGAGCC-BFQ-3′

primer forward RPL13A 5′-GGCAATTTCTACAGAAACAAGTTG-3′

primer reverse RPL13A 5′-GTTTTGTGGGGCAGCATACC-3′

probe RPL13A 5′-HEX-CGCACGGTCCGCCAGAAGAT-BFQ-3′

primer forward β-actin 5′-ACAGAGCCTCGCCTTTG-3′

primer reverse β-actin 5′-ACGATGGAGGGGAAGACG-3′

probe β-actin 5′-Cy5-CCTTGCACATGCCGGAGCC-BRQ-3′

primer forward GAPDH 5′-ACATCGCTCAGACACCATG-3′

primer reverse GAPDH 5′-TGTAGTTGAGGTCAATGAAGGG-3′

probe GAPDH 5′-FAM-AAGGTCGGAGTCAACGGATTTGGTC-BFQ-3′

**Supplementary Figure S1.**

**
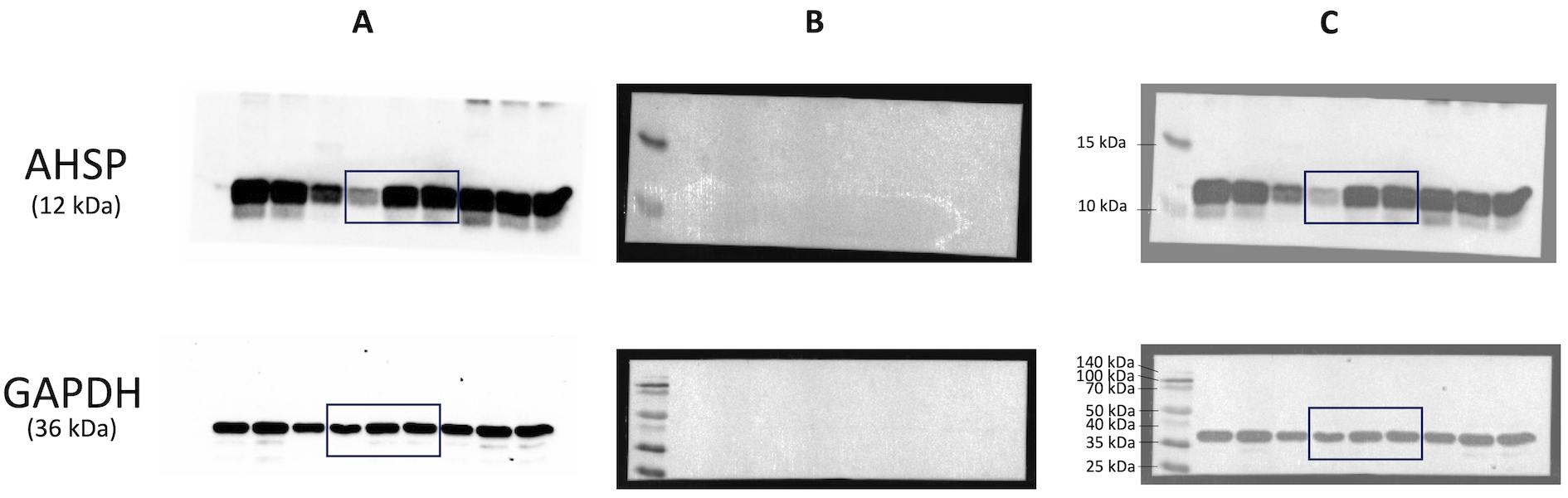
**

**Figure S1.** Uncropped version of Western Blot presented in Figure 1A. In panel A we show the acquired blot image (boxed), in panel B the nitrocellulose membrane with the pre-stained multicolor protein ladder (Spectra pre-stained ladder by Thermo Fisher, Waltham, MA, USA, cat. n. 26634) and in panel C the merge of picture A and B, showing the exact molecular weight of the target proteins.

**Materials and Methods**

**1. Western Blotting (*)**

In order to measure changes in AHSP protein content, AHSP was quantified in ErPC cytoplasmic extracts by Western Blotting. Protein concentration in these extracts was determined using PierceTM BCA Protein Assay Kit (Thermo Fisher, Waltham, MA, USA), before to run the gels. Twenty μg of cytoplasmic extracts were denatured for 5 min at 98°C in SDS sample buffer (Cell Signalling Technology, Danverss, MA, USA), and loaded on hand casted SDS-PAGE 14% gel (10 cm × 8 cm) in Tris-glycine Buffer (Bio-Rad, Hercules, California, USA). Then, electro-transfer to 0.2 μm pore size nitrocellulose membrane (Thermo Fisher, Waltham, MA, USA) was performed over-night at 360 mA and 4°C in standard Tris-Glycine-MeOH transfer buffer. After washing in TBS-T, the membranes were incubated with the primary antibodies. The different phases of the methods are described in Zurlo et al. [1]. For analysis of the blots images the Bio-Rad Image Lab Software (Bio-Rad, Hercules, California, USA) was used. The employed antibodies are reported in **Table S3.**

**Table S3.** Western blot primary and secondary antibodies employed for detection of protein present in ErPCs lysates

| **Target** | **Primary antibody** | **Cat.n.** | **Secondary antibody** | **Cat.n.** |
| --- | --- | --- | --- | --- |
| AHSP | Rabbit anti-AHSP  (ABclonal, Woburn, MA, USA) | A6465 | Mouse Anti-rabbit IgG HRP  (Cell Signalling Technology,  Danvers, MA, USA | 7074 |
| GAPDH | Mouse anti-GAPDH  (Thermo Fisher,  Waltham, MA, USA) | MA1-16783 | Goat Anti-mouse IgG HRP  (Thermo Fisher,  Waltham, MA, USA) | 32430 |

**RT-qPCR analysis (*)**

In order to measure changes in *AHSP* and Ulk-1 gene expression, these mRNAs were quantified by Reverse Transcription-quantitative-Real Time PCR (RT-qPCR). The total cellular RNA was extracted by using TRI Reagent® (Sigma-Aldrich, Saint Louis, Missouri, USA), as elsewhere described [1,2].

For gene expression analysis, 300 ng of total RNA was reverse transcribed using the PrimeScript RT kit from Takara Bio (Takara Bio Inc., Shiga, Japan), as recently reported [1,2]. To quantify the expression of the *AHSP* and *Ulk-1* genes, quantitative real time PCR assay (RT-qPCR) was performed, and the content of *AHSP* and *Ulk-1* mRNAs was compared to the house-keeping sequences *GAPDH*, *RPL13A* and *β-actin* (all probes and primers are reported in **Table S2**).

Each reaction mixture contained 1x TaKaRa Ex Taq® DNA Polymerase (Takara Bio Inc., Shiga, Japan). 300 nM PCR primers and 200 nM probes (Integrated DNA Technologies, Castenaso, Italy) were employed using CFX96 Touch Real-Time PCR System (Bio-Rad, Hercules, California, USA). The following protocol was used: initial denaturation at 95°C, 1 min; 50 PCR cycles performed (95°C for 15 sec, 60°C for 60 sec). The CFX manager software (Bio-Rad, Hercules, California, USA) was employed for data analysis using the ΔΔCt method for quantification [1,2].

**References**

1. Zurlo M, Zuccato C, Cosenza LC, Gamberini MR, Finotti A, Gambari R (2024) Increased Expression of α-Hemoglobin Stabilizing Protein (AHSP) mRNA in Erythroid Precursor Cells Isolated from β-Thalassemia Patients Treated with Sirolimus (Rapamycin). J Clin Med 13(9):2479..

2. Zurlo, M.; Zuccato, C.; Cosenza, L.C.; Gasparello, J.; Gamberini, M.R.; Stievano, A.; Fortini, M.; Prosdocimi, M.; Finotti, A.; Gambari, R. Decrease in α-Globin and Increase in the Autophagy-Activating Kinase ULK1 mRNA in Erythroid Precursors from β-Thalassemia Patients Treated with Sirolimus. Int. J. Mol. Sci. 2023, 24, 15049.

(*) The methods for Western blotting and RT-qPCR analysis were from Zurlo et al. [1] and Zurlo et al. [2]. These articles are open access articles distributed under the terms and conditions of the Creative Commons Attribution (CC BY). The copyright and license information can be found at: https://pmc.ncbi.nlm.nih.gov/articles/PMC11084795/ and https://www.mdpi.com/1422-0067/24/20/15049.
